# Supplementary material for: Synovial Membrane Is a Major Producer of Extracellular Inorganic Pyrophosphate in Response to Hypoxia
Source: Pharmaceuticals (Basel). 2024 Jun 5;17(6):738. doi: 10.3390/ph17060738 (PMC11206467; doi:10.3390/ph17060738)
Supplement: Supplementary file 1 [file pharmaceuticals-17-00738-s001.zip › Supplementary S1 _Material_Pharmaceuticals_Bianchi et al 2024.pdf]

Synovial Membrane Is a Major Producer of Extracellular Inorganic Pyrophosphate in Response to Hypoxia

Émilie Velot, Sylvie Sébillaud and Arnaud Bianchi *

Université de Lorraine, CNRS, IMoPA, F-54000 Nancy, France; emilie.velot@univ-lorraine.fr (É.V.); sylvie.sebillaud@inrs.fr (S.S.)

* Correspondence: arnaud.bianchi@univ-lorraine.fr

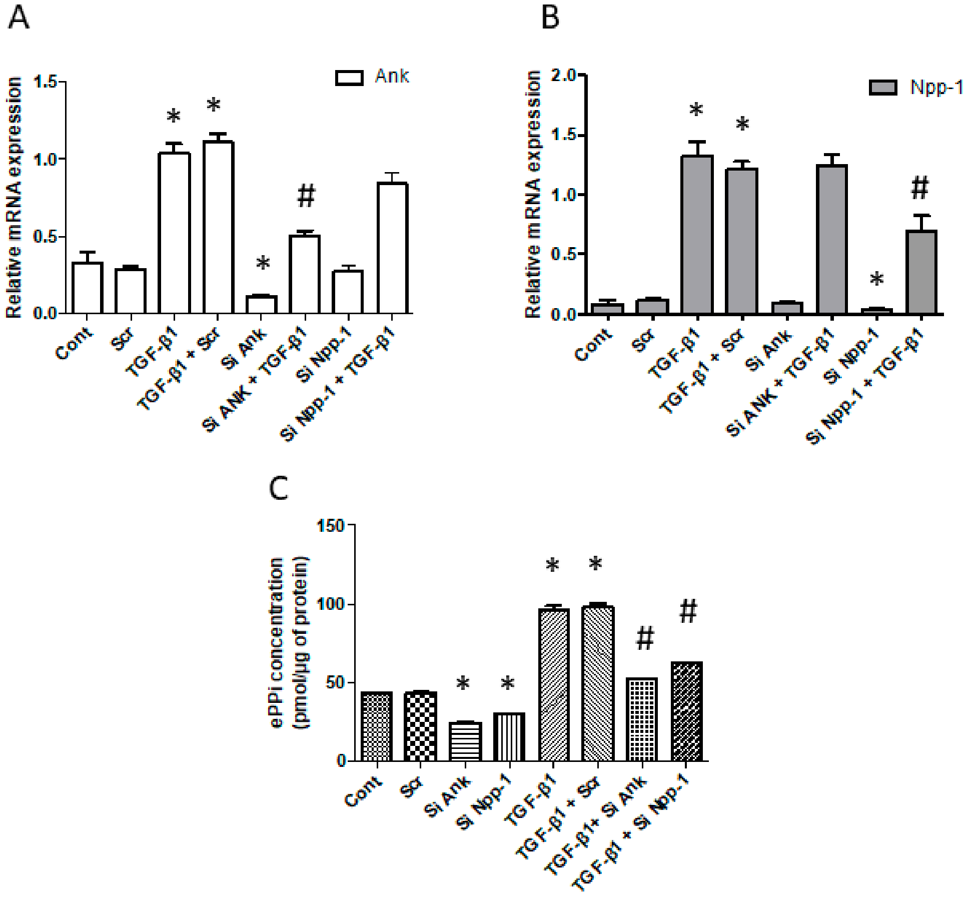

Supplementary Figure S1. *Ank* contributes more than *Enpp1* to the TGF-β1-induced production of ePPi *in vitro*. **(A to C)** Synovial fibroblasts cultured in monolayer were transfected or not with scramble siRNA or siRNAs directed against *Ank* or *Enpp1* for 24 h before being challenged or not with 10 ng/mL of TGF-β1 for 24 h. Total RNA was extracted from cells and subjected to RT-qPCR analysis. The abundance of *Ank* **(A)** and *Enpp1* **(B)** mRNA was normalized to that of *Rp29* mRNA. Results are expressed as means (± SD) over *Rp29* values. **(C)** Levels of ePPi were assessed in culture supernatant of synovial fibroblasts

transfected or not as mentioned above, then stimulated or not with 10 ng/mL of TGF-β1 for 48 h. ePPi levels were normalized to the amount of total cell proteins and are expressed as mean (± SD) in picomoles per microgram of protein. Statistically significant differences are indicated as * for $p < 0.05$ from the control and as # $p < 0.05$ from TGF-β1-stimulated cells. (*Ank*: inorganic pyrophosphate transport regulator; Cont: control condition meaning no TGF-β1 treatment and no transfection; *Enpp1*: ectonucleotide pyrophosphatase/phosphodiesterase 1; ePPi: extracellular inorganic pyrophosphate; *Rp29*: ribosomal protein 29; RT-qPCR: reverse transcription–quantitative polymerase chain reaction; Scr: scramble RNA meaning non-silencing RNA; SD: standard deviation; Si: means siRNA or small interfering RNA; TGF: transforming growth factor).
